# Large Population Analysis of Secondary Cancers in Pediatric Leukemia Survivors

**DOI:** 10.3390/children6120130

**Published:** 2019-11-29

**Authors:** Siddhartha Yavvari, Yasaswi Makena, Sahithi Sukhavasi, Monish Ram Makena

**Affiliations:** 1 Department of Epidemiology, Johns Hopkins Bloomberg School of Public Health, Baltimore, MD 21205, USA; syavvar1@jhu.edu; 2 Department of Biotechnology, GITAM University, Visakhapatnam, AP 530045, India; yasaswimakena33@gmail.com; 3 Center for Distance Learning, GITAM University, Visakhapatnam, AP 530045, India; Sahithisukhavasi1695@gmail.com; 4 Department of Physiology, Johns Hopkins School of Medicine, Baltimore, MD 21205, USA

**Keywords:** leukemia, second malignant neoplasms, pediatric cancers, SEER

## Abstract

Introduction: Survivors of childhood cancer have an increased risk of developing a subsequent secondary malignant neoplasm (SMN). Among five-year survivors of primary cancer, SMNs account for nearly half of non-relapse deaths, which make them the most frequent cause of non-relapse mortality. Leukemia is the most common childhood cancer and the five-year survival rate of leukemia has drastically improved over the past two decades. Therefore, the chances of developing SMNs are higher in pediatric (0–19 years) leukemia survivors. Methods: The US based Surveillance, Epidemiology, and End Results (SEER-18) database (1973–2014) was probed for SMNs in the pediatric population (age ≤ 19). Variables Sequence-number central, primary site and ICCC3WHO were used to identify the first and second cancers among patients who developed SMN. Results: Our SEER database analysis found 99,380 cases of pediatric primary malignancies (0–19 years), of which 1803 (1.81%) patients developed SMN. The breakdown of SMNs in pediatric leukemia survivors (n = 251) showed thyroid carcinoma (18.33% of cases) as the most common second cancer, followed by sarcoma (15.14%), astrocytoma (10.36%), lymphoma (9.56%), salivary gland carcinoma (7.17%), melanoma (4.38%), and breast cancer (3.98%). Interestingly, we found that over 76% of SMNs that were developed by leukemia patients occurred within 20 years after initial leukemia diagnosis. However, some SMNs occur during later age, for example, the mean age for breast cancer occurrence in leukemia survivors is 26.20 ± 8.53 years after initial leukemia diagnosis. Conclusions: Our study presented comprehensive rates of SMNs among pediatric cancers survivors, and the potential SMNs for pediatric leukemia survivors. This information could we used by oncologists, patients, patient families, and cancer researchers to understand the long-term risks that are associated with the development of SMNs in pediatric leukemia survivors.

## 1. Introduction

Cancer is the second most common cause of death among children (1–14 years) in the United States. In 2019, it is estimated that 11,060 children will be diagnosed with cancer and 1190 will eventually die from the disease [1]. There has been a decline in cancer mortality in children and adolescents (15–19 years) over the past decades. Cancer mortality was reduced from 6.5 per 100,000 population in 1970, to 2.3 per 100,000 population in 2016 [1]. This reduction is approximately 65% in children and 61% in adolescents [1]. Survival after childhood cancer exceeds 80% in the United States and Europe [2,3]. The number of pediatric cancer survivors in the United States is predicted to approach 500,000 by 2020, which is approximately one survivor for every 750 individuals [4]. As the survival of the pediatric patients (0–19 years) continues to improve, the burden of long-term effects of therapies is also on rise. Secondary malignant neoplasm (SMN), which are new primary malignancies after an initial cancer diagnosis, are one of the side effects of cancer therapy [4]. Among five-year survivors of primary cancer, SMNs account for nearly half of non-relapse deaths, which makes them the most frequent cause of non-relapse mortality [5]. Twenty years following the original diagnosis, pediatric cancer survivors have a four-to-six fold greater risk of developing a new cancer than the general population [5]. Therefore, SMNs are an increasing epidemiologic concern in pediatric cancer survivors.

Risk factors that lead to SMNs include age, hereditary predisposition, and side effects of cancer therapy while treating the first malignancy [6]. Single malignant cell forms tumor with the passage of time, thus the diagnosis of SMNs increases with survivorship years. Genetic or hereditary risk factors, such as RB1, BRCA1/2, NF1, TP53, MLH1, MSH2, MSH6 VHL47, DICER1, and etc., are associated with SMNs [6,7]. Alkylating agents that are used to treat many pediatric malignancies increase the relative risk for a SMN by 1.4–2.2 fold. In particular, exposure to alkylating agents is associated with an increased risk of hematologic malignancies [6,7]. The exposure of first malignancies with topoisomerase inhibitors have an increased risk of developing solid tumors and acute myeloid leukemia as SMNs [6]. Ionizing radiation is a part of standard care therapy for many childhood cancers. Therapy-related ionizing radiation exposure was associated with a 2.7-fold relative risk of developing SMNs, and it is the strongest independent risk factor in the development of SMNs [6]. Several pediatric cancers are preconditioned with Hematopoietic Stem Cell Transplantation (HSCT) before the treatment; patients undergoing HSCT are reported to have an eight-fold higher incidence of SMNs as compared to the general population [6]. Further, environmental/behavioral factors are also associated with SMNs [6].

Leukemia is a cancer of the early blood-forming cells. Leukemia are broadly classified as acute (fast growing) or chronic (slower growing), and by whether they originate from myeloid cells or lymphoid cells [8]. Leukemia is the most common childhood cancer, constituting 29% of all pediatric cancers, 22% of these are lymphoid leukemia, and 4% are myeloid leukemia. In adolescents, leukemia is the third most common cancer, accounting for 13% of total cases [1].

The five-year overall survival rate of lymphoid leukemia is over 90% in children and close to 75% in adolescents, and ~65% in myeloid leukemia [1,9,10]. This means that many pediatric patients with leukemia will live longer. Further, the treatment of both lymphoid and myeloid leukemia include alkylating agents, radiation, and stem cell transplant is used sometimes to improve the chances of cure [8].

Taken together, these factors contribute to elevated chances of SMNs in pediatric leukemia survivors. Therefore, we probed The Surveillance, Epidemiology, and End Results (SEER) database to report the incidence of SMNs in pediatric leukemia survivors.

## 2. Materials and Methods

### SEER Database Analysis

We utilized the Surveillance, Epidemiology, and End Results (SEER) database, which includes data from 18 cancer registries (SEER-18) and represents 28% of the US population, to extract the study population. Pediatric patients (age ≤ 19) at the time of diagnosis of first malignancy who developed a second cancer, excluding relapse, between the years 1973–2014 were selected. The patients who were reported as having non-malignant tumors and those with unknown age were excluded from the analysis. We used the Sequence number-central variable to identify the patients who developed more than one reportable malignant neoplasm. We used the variables primary site and ICCC3WHO (International Classification of Childhood Cancer, World Health Organization) (ICCC Site Recode ICD-O-3/ WHO 2008), a recoded variable provided by SEER that is based on site/histology, to exclude the cases with the same values in the first and second cancers. Further, we created broader categories to identify and exclude cases with different ICCC3WHO recodes but similar malignancies. For example, a patient reported developing lymphoid leukemia as the first cancer and myeloid leukemia as the second cancer was excluded from the analysis. Further, we identified the number of pediatric patients (0–19 years) with leukemia who developed second cancers. The median follow up time in the SEER database is 12 years (range: 0–41) for patients who developed second cancers, and 13 years (range: 0–41) for leukemia survivors who developed SMN.

## 3. Results

Our SEER database analysis found that, out of 99,380 cases of primary pediatric malignancies (0-19 years), 24,403 cases (24.58%) are leukemia. The total number of SMN cases developed is 1803 (1.81%). The most common SMN was leukemia, which accounted for 227 cases (12.59% of total cases). The second most common SMN is thyroid cancer (217, 12.04%), followed by sarcoma (207, 11.48%), breast carcinoma (203, 11.26%), malignant melanoma (89, 4.94%), and lymphoma (83, 4.60%) (Table 1, Figure S1A).

**Figure 1:**
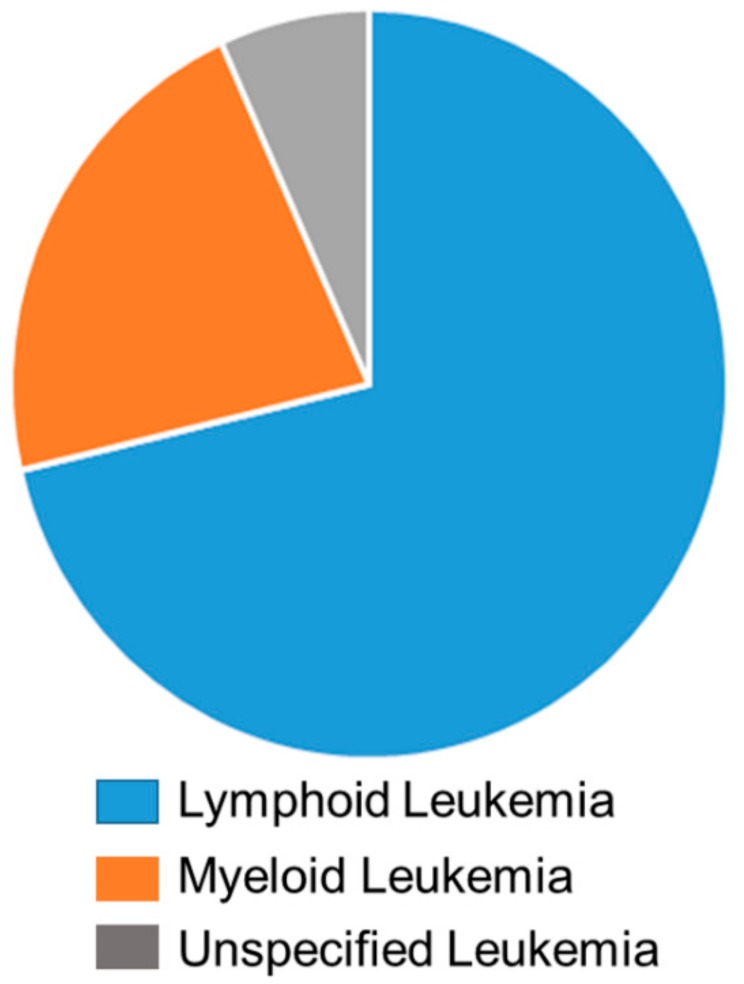
The breakdown of primary leukemia into sub-groups. Lymphoid leukemia are represented in blue (71.31%), myeloid leukemia are represented in orange (21.91%), and unspecified and specified leukemia are represented in grey (6.78%).

Out of 24,403 leukemia cases, 251 patients (1.03% of total leukemia cases) developed secondary cancers. The breakdown of leukemia primary cancer into the sub groups showed that lymphoid leukemia patients are more prone to second cancer, accounting for 179 cases (71.31%). Fifty-five cases (21.91%) were reported as myeloid leukemia, and 17 cases (6.78%) are unspecified and specified leukemia (Figure 1).

The breakdown of SMNs in pediatric leukemia survivors showed that thyroid carcinoma is the most common second cancer, where 46 cases are reported, accounting for 18.33%, which is approximately one-fifth of all SMNs. Sarcoma is the second most common, with 38 cases (15.14%), followed by astrocytoma (26 cases, 10.36%), lymphoma (24 cases, 9.56%), salivary gland carcinoma (18 cases, 7.17%), melanoma (11 cases, 4.38%), breast cancer (10 cases, 3.98%), and etc. (Table 2, Figure S1B).

Time since diagnosis is one of the factors for SMNs, and we investigated the effect of elapsed time for subsequent cancers after leukemia. Interestingly, the incidence of SMNs decreases with age in pediatric leukemia survivors. We found that 43.42% (109 of 251 cases) SMNs developed within 10 years after primary diagnosis of leukemia and 76.09% (191 of 251 cases) SMNs occurred within 20 years (Table 3).

We also analyzed the distribution of sex, age at initial diagnosis, and cases by decade for the pediatric leukemia survivors who developed SMNs. There is no sizable difference in the number of males and females developing SMNs in lymphoid leukemia, females are more prone to SMNs in myeloid leukemia (Table 4).

## 4. Discussion

SMNs are rare but devastating complications for cancer survivors. The five-year survival rate of pediatric leukemia has seen significant improvement over the last two decades. Further, leukemia treatment includes alkylating agents and radiation. This means that many pediatric patients with leukemia will live longer and have an increased chance of developing SMNs. Our study showed that leukemia is the most common SMN in pediatric survivors. Here, we report 251 pediatric leukemia survivors who were diagnosed with SMNs, where thyroid cancer is the most common SMN, followed by sarcoma, astrocytoma, and lymphoma.

Our results show that leukemia is a common SMN in the pediatric population. Similar results were also shown in a retrospective review that was conducted on patients diagnosed (1990–2012) with childhood cancer under age 21 in National University Hospital, Singapore [11]. Analysis of medical data in the Korea Central Cancer Registry (KCCR) showed leukemia as the second most common SMN in Korean childhood cancer patients (diagnosed from 1993–2012) [12]. The Pediatric Oncology Group of Ontario Networked Information System (POGONIS) captures 98% of the incident cancer cases in Ontario that were diagnosed in the 0 to 14.9-year age range. Analysis of SMNs in the POGONIS databased showed that 40% of SMNs developed within five years after primary cancer, and leukemia was the most common SMN accounting for 23.9% of total SMNs [13]. Nottage, K. et al. reported that there is a statistically significantly increased risk of subsequent leukemia occurring ≥15 years from treatment of a primary childhood cancers [14]. These results show a trend that is consistent with our results.

Similar to our results, thyroid cancer was reported as the second most common (22% of total cases) SMN in patients that were diagnosed with acute myeloid or acute lymphoblastic leukemia (0–18 years), who survived at least five years after diagnosis [15]. Similarly, a population-based registry study in which 13 registries from around the world provided individual data on more than 16,500 survivors of childhood leukemia showed that lymphoma and thyroid cancers are among the top three SMNs [16]. Another study obtained chemotherapy and radiotherapy information from the medical records of 12,547 five-year survivors of childhood cancer diagnosed from 1970 through 1986, showed that treatments with alkylating agents increased the chance of developing thyroid cancer SMNs [17]. Childhood acute leukemia survivors who received radiotherapy were diagnosed with the greatest risk of short stature, hypothyroidism, and a reduced likelihood of pregnancy or live birth [18]. Additionally, various sarcoma cases are reported as SMNs in pediatric leukemia survivors [19,20,21].

Similar to our study, Choi DK et al. reported that SMNs decrease in childhood cancer survivors with the increase in time elapsed since primary malignancy diagnosis [6]. However, the mean age for development of breast cancer SMNNMs in pediatric leukemia survivors is 26.2 ± 8.53 years. Similarly, it is 19.6 ± 9.67 years for thyroid cancer SMNs. This shows the time elapsed since primary diagnosis is still an important determinant of SMNs for some cancers.

Adolescents and young adults who survive cancer for more than five years were shown to have a higher relative risk of SMN as compared with the general population, and they have a higher absolute risk of SMN when compared with younger or older cancer survivors [22,23].

Although the SEER database is an extremely valuable tool it has a few limitations. SEER data does not track long term outcomes, toxicities, and the cumulative chemotherapy doses of primary cancers. With regard to the investigation of SMNs, a patient moving away from a SEER registry region would be lost from the database, and a subsequent SMN would be unrecorded. This might lead to a relative undercounting of SMNs [24]. A limitation of our study is the exclusion of survivors who suffered from two distinct leukemias. In our analysis, we defined SMN as developing a different second cancer when compared to primary. As a result, a total of 100 patients with leukemia were excluded because they developed leukemia as both primary and secondary cancer. Among them, twelve patients had same type of leukemia and, in 27 cases, diagnosis is unspecified in either primary or secondary leukemia. The remaining 61 excluded cases include patients with distinct leukemia as primary and secondary cancers (for example, myeloid leukemia as primary and lymphoid leukemia as secondary cancer respectively).

## 5. Conclusions

Our study presented comprehensive rates of SMNs among pediatric cancers survivors, and the potential SMNs for pediatric leukemia survivors. This information will be helpful for oncologists, patients, patient families, and cancer researchers to understand the long-term risks that are associated with the development of SMNs in pediatric leukemia survivors.

## Figures and Tables

**Table 1:** Secondary malignant neoplasms (SMNs) in Pediatric Cancer Survivors. SMN distribution in pediatric cancer survivors (0–19 years), analyzed using SEER database.

Cancer	No. of Cases	% of Total SMN
Leukemia	227	12.59
Thyroid	217	12.04
Sarcoma	207	11.48
Breast	203	11.26
Melanoma	89	4.94
Lymphoma	83	4.60
Colon and Rectum	63	3.49
Astrocytoma	56	3.11
Renal	46	2.55
Lung	43	2.38
Others	569	31.56
Total	1803	100%

**Table 2:** SMNs in Pediatric Leukemia Survivors. SMN distribution in pediatric leukemia survivors, analyzed using SEER database.

Cancer	No. of Cases	% of Total SMN
Thyroid	46	18.33
Sarcoma	38	15.14
Astrocytoma	26	10.36
Lymphoma	24	9.56
Salivary gland carcinoma	18	7.17
Melanoma	11	4.38
Breast	10	3.98
Others	78	31.08
Total	251	100%

**Table 3:** SMNs Distribution by Difference in Time Elapsed Since Diagnosis in Leukemia Survivors. Distribution of SMN occurrence by time elapsed since diagnosis of primary leukemia, analyzed using SEER database.

Difference in Years	No. of Cases	% of Total SMN
0–5	56	22.30
06 to 10	53	21.12
11 to 15	45	17.93
16 to 20	37	14.74
21 to 25	20	7.97
26 to 30	14	5.58
31 to 35	19	7.57
>36	7	2.79
Total	251	100%

**Table 4:** Distribution by Sex, Age at Primary Diagnosis, and Cases by Decade in Leukemia Survivors, analyzed using SEER database. LL—Lymphoid leukemia, ML—Myeloid leukemia, and UL- Unspecified leukemia.

	LL (n = 179)	ML (n = 55)	UL (n = 17)
**Sex**			
Male	93 (52.0%)	21 (38.2%)	7 (41.2%)
Female	86 (48.0%)	34 (61.8%)	10 (58.8%)
**Age at Primary Diagnosis (Years)**			
Median (IQR)	5 (3–11)	9 (4–15)	6 (3–9)
**Cases by Decade**			
1973–1982	45	13	7
1983–1992	45	12	3
1993–2002	57	14	3
2003–2013	32	16	4

## Supplementary Materials

All the supplementary data for this article are available online at https://www.mdpi.com/2227-9067/6/12/130/s1. Figure S1; Flow chart representing Table 1 (Panel A) and Table 2 (panel B).
