# Supplementary figures and images for: Large Population Analysis of Secondary Cancers in Pediatric Leukemia Survivors
Source: Children (Basel). 2019 Nov 29;6(12):130. doi: 10.3390/children6120130 (PMC6956149; doi:10.3390/children6120130)

**Figure S1:** Flow chart representing Table 1 (Panel A) and Table 2 (panel B).

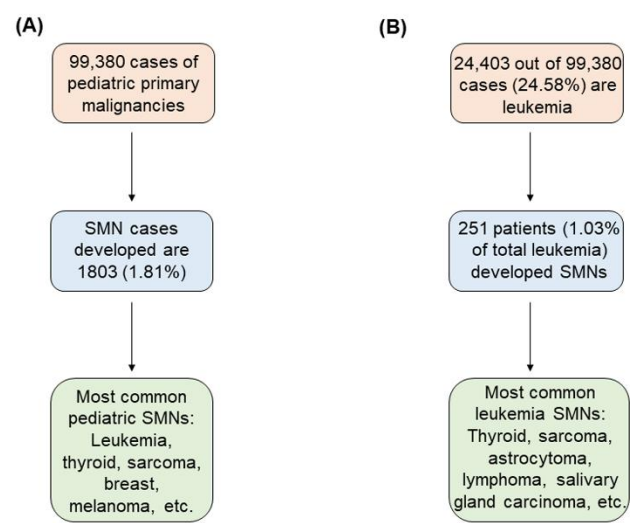

Supplement: Supplementary file 1 [file children-06-00130-s001.pdf]
